# Pediatric Blast Trauma: A Systematic Review and Meta-Analysis of Factors Associated with Mortality and Description of Injury Profiles

**DOI:** 10.1017/S1049023X22000747

**Published:** 2022-08

**Authors:** Matthew A. Tovar, Rebecca A. Pilkington, Tress Goodwin, Jeremy M. Root

**Affiliations:** 1. School of Medicine and Health Sciences, George Washington University, Washington, DC USA; 2. School of Medicine, Virginia Commonwealth University, Richmond, Virginia USA; 3. Emergency Medicine and Trauma Services, Children’s National Hospital, Washington, DC USA

**Keywords:** blast injuries, Disaster Medicine, disaster planning, mass-casualty incidents, pediatric emergency medicine

## Abstract

**Introduction:**

Blast polytrauma is among the most serious mechanisms of injury confronted by medical providers. There are currently no specific studies or guidelines that define risk factors for mortality in the context of pediatric blast injuries or describe pediatric blast injury profiles.

**Objective:**

The objectives of this study were to evaluate risk factors for pediatric mortality and to describe differences in injury profiles between explosions related to terrorism versus unrelated to terrorism within the pediatric population.

**Methods:**

A PRISMA systematic review and meta-analysis was performed where articles published from the years 2000-2021 were extracted from PubMed. Mortality and injury profile data were extracted from articles that met inclusion criteria. A bivariant unadjusted odds ratio (OR) analysis was performed to establish protective and harmful factors associated with mortality and to describe the injury profiles of blasts related to terrorism. Statistical significance was established at P < .05.

**Results:**

Thirty-eight articles were included and described a total of 222,638 unique injuries. Factors associated with increased mortality included if the explosion was related to terrorism (OR = 32.73; 95% CI, 28.80-37.21; P < .05) and if the explosion involved high-grade explosives utilized in the Global War on Terror ([GWOT] OR = 1.28; 95% CI, 1.04-1.44; P < .05). Factors associated with decreased mortality included if the patient was resuscitated in a North Atlantic Treaty Organization (NATO)-affiliated combat trauma hospital (OR = 0.48; 95% CI, 0.37-0.62; P < .05); if the explosive was fireworks (OR = 3.20×10-5; 95% CI, 2.00×10-6-5.16×10-4; P < .05); and if the explosion occurred in the United States (OR = 2.40×10-5; 95% CI, 1.51×10-6-3.87×10-4; P < .05). On average, victims of explosions related to terrorism were 10.30 years old (SD = 2.73) with 68.96% (SD = 17.58%) of victims reported as male. Comparison of victims of explosions related to terrorism revealed a higher incidence of thoracoabdominal trauma (30.2% versus 8.6%), similar incidence of craniocerebral trauma (39.5% versus 43.1%), and lower incidence of extremity trauma (31.8% versus 48.3%) compared to victims of explosions unrelated to terrorism.

**Conclusion:**

Explosions related to terrorism are associated with increased mortality and unique injury profiles compared to explosions unrelated to terrorism in the pediatric population. Such findings are important for optimizing disaster medical education of pediatric providers in preparation for and management of acute sequelae of blast injuries—terror-related and otherwise.

## Introduction

Children are among the most vulnerable populations inadvertently affected by explosions both related and unrelated to terrorism. For the last two decades, the primary battlespace of war and civil conflict continues to be crowded cities and urban environments. Thus, children are killed and injured either intentionally or collaterally by explosive weapons. This has been evident in modern-day conflicts in Iraq,^1^ Afghanistan,^2^ Syria,^3^ and Gaza.^4^ Parallel to the threat of terrorism and counter-terrorism warfare, accidental explosions are another potential means causing pediatric civilian casualties. This was most recently seen in the 2020 explosion in Beirut, Lebanon where over 200 people were killed (including six children) and tens of thousands more were injured (including over 1,000 children) by ammonium nitrate incorrectly stored adjacent to a fireworks factory.^5^ On a smaller scale, injuries secondary to accidental fireworks explosions also have the potential to produce significant blast injury in the pediatric population.^6–8^ Natural gas explosions and accidental detonation of old warfare ordnance are additional causes of pediatric blast injuries outside of armed conflict. Given that there have been, on average, 3,400 bombings per year since 2000^9^ and over 15,000 fireworks-related injuries in the United States in the year 2020 alone,^10^ it is clear that the medical enterprise must be well-prepared to successfully triage and resuscitate pediatric patients who have experienced devastating blast injuries. To date, no studies have directly analyzed environmental risk factors for pediatric mortality in blast trauma or differences in pediatric injury patterns based on if explosions were related or unrelated to terrorism. Such data would be useful to design subsequent training for emergency medical personnel and to prepare medical organizational disaster response plans. Therefore, the objective of this study was to analyze risk factors for pediatric mortality and injury profiles in the setting of explosions related and unrelated to terrorism.

## Methods

### Literature Search

The systematic review was carried out under Preferred Reporting Items for Systematic Reviews and Meta Analyses (PRISMA) guidelines (Supplementary Material, Table S1; available online only) and was designed to answer the following Population, Intervention, Comparison, Outcome (PICO)-centered question: Is there a difference in mortality and blast injury patterns (outcome) between pediatric patients (population of interest) affected by explosions related to terrorism (exposure) and pediatric patients affected by explosions unrelated to terrorism (comparison)? PubMed (National Center for Biotechnology Information, National Institutes of Health; Bethesda, Maryland USA) was used as the primary search engine, and the following search terms were utilized*: “child”[MeSH Terms] OR “child”[All Fields] OR “children”[All Fields] OR “child s”[All Fields] OR “children s”[All Fields] OR “childrens”[All Fields] OR “childs”[All Fields]) AND (“blast injuries”[MeSH Terms]) OR (“blast”[All Fields] AND “injuries”[All Fields]) OR “blast injuries”[All Fields] OR (“blast”[All Fields] AND “injury”[All Fields]) OR “blast injury”[All Fields]*. All articles were subject to a screening protocol where two independent reviewers screened the full text against pre-set inclusion and exclusion criteria. To be considered for inclusion, the text had to have been written in the English language and published after the year 2000. The text also had to have provided an injury profile, defined as a list of injuries sustained from an explosion stratified by Abbreviated Injury Scale (AIS) body region. The AIS is an anatomically based scoring system that describes injury by body region; it is the basis for the Injury Severity Score (ISS) utilized to assess overall severity in the patient with systemic polytrauma. The AIS codes are stratified anatomical areas and include the head, face, and neck (AIS Regions 1-3), thorax (AIS Region 4), abdomen (AIS Region 5), and upper and lower extremities (AIS Regions 7-8). In addition, articles must have reported the injury profiles of pediatric patients where “pediatric” was defined in this study as any patient less than age 18. If articles reported both adult and pediatric injury profiles, articles were included only if pediatric patients had an injury profile separate from the adult population. Exclusion criteria included basic science research, quality improvement studies, studies not including pediatric patients, studies describing injuries focused on one part of the body (for example, a study solely describing blast-related ophthalmologic injury), case studies, and any study not published in a peer-reviewed journal. Studies with missing data were excluded *post hoc.* Finally, the references of articles meeting the present inclusion criteria were screened for additional relevant articles not captured by the initial data extraction. The systematic review protocol was carried out by two authors (MT, RP) with any discrepancies decided on by a third author (JR).

### Data Quality Analysis

All studies were appraised for scientific rigor using an 11-point modified Mixed Method Appraisal Tool (MMAT)^11^ with discreet scores given of -1, 0, and +1 where articles fully meeting a given listed quality metric are graded at +1, articles where not meeting the quality metric cannot be ruled out are graded at 0, and articles definitively not meeting the quality metric are graded at -1. The quality metric grades were then summated for each article and are reported in Supplementary Material, Table S2 (available online only). Questions on the MMAT utilized in this work were those pertaining to quantitative non-comparative descriptive studies. Each question on the MMAT was formulated and adapted to relate to metrics relevant to this study. Data quality analysis was performed by two researchers (MT, RP).

### Data Extraction and Analysis

Injury pattern data were extracted from each study and stratified into AIS-defined body regions. Other data extracted included the type of study, years analyzed, geographic location of the explosion(s), mean casualty age, biological sex distribution, mortality rate, total number of patients studied, ISS, and if the explosion was deemed to be related or unrelated to terrorism. The definition of terrorism utilized here was derived from the United States Code of Federal Regulations (28 C.F.R. § 0.85). An explosion was defined as being related to terrorism if the act occurred with the intent of producing immediate casualties and the assailants had the political and/or social objective of instilling communal fear. If an event did not meet this criterion, it was deemed to be unrelated to terrorism.^12^

All statistical analysis was performed in the GraphPad Prism statistical software package (version 09; GraphPad Software; San Diego, California USA). Data sets were initially examined for normality using four independent normality tests: the Anderson-Darling test,^13^ D’Agostino-Pearson test,^14,15^ Shapiro-Wilk test,^16^ and Kolmogorov-Smirnov test.^17^ In addition, QQ plots of each of the four injury pattern data sets were analyzed. Continuous data sets that passed all four quantitative tests for normality in addition to qualitative QQ plot analysis were analyzed for statistical significance using Welch’s t test of unequal variance.^18^ Welch’s t test was selected over student’s t test as it provides better control of Type I errors when homoscedasticity (the assumption that data variance is homogenous) is violated, while performing as well as the student’s t test when homoscedasticity is maintained.^19–22^ Aggregated data were reported as mean values with data spread described as standard deviation (SD). Discreet data were collated into contingency tables to ascertain bivariate odds ratios (OR) with variables being represented as a hypergeometric probability function of explosions related versus unrelated to terrorism. An exact test approach was chosen over the chi-squared approximation as the latter relies on the assumption that the central limit theorem is met, while the former does not and was thus posited by the authors to be of higher reliability.^23^ Fisher’s exact test was selected over Barnard’s exact test given the equivalent power generation of both tests at high sample numbers coupled with the more conservative determination of significance associated with Fisher’s exact test.^24–26^ This results in a deliberately higher sensitivity in establishing statistical significance with Fisher’s test as compared to Barnard’s test. The 95% odds ratio confidence intervals (CI) were calculated using the Woolf-Logit models. Statistical significance was conferred if P < .05.

## Results

### Systematic Review

**Figure 1.**
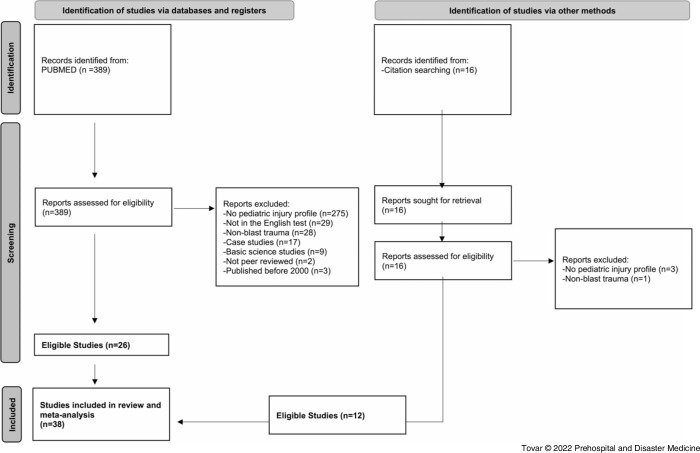
A 2020 PRISMA Flow Diagram of the Systematic Review Process Performed to Select Articles for Inclusion into Study. Abbreviation: PRISMA, Preferred Reporting Items for Systematic Reviews and Meta Analyses.

A total of 389 articles were extracted from PubMed using the search criteria previously described. Twenty-six studies from the initial data extraction were found to meet inclusion criteria. Twelve additional studies that met inclusion criteria were identified by searching the citations from the original articles (Figure 1). Thus, a total of 38 studies were included in the systematic review and meta-analysis, as shown in Table 1.^27–64^ The most common causes for article exclusion were no pediatric injury profiles included (n = 275; 75.9%); articles not written in English (n = 29; 8.0%); articles did not describe blast injuries (n = 28; 7.7%); articles identified as case studies (n = 17; 4.7%); articles identified as basic science studies (n = 9; 2.49%); non-peer-reviewed studies (n = 2; 0.55%); and articles describing events not in the specified date range (n = 3; 0.83%; Figure 1).

**Table 1.** Summary of Studies Investigating Pediatric Blast Trauma

	PMID	Authors	Year Published	Country	Study Period
1	10923857	CDC, et al^27^	2000	USA	1997-2000
2	11259737	Terzić J, et al^28^	2001	Croatia	1991-1995
3	14523212	Aharonson-Daniel L, et al^29^	2003	Israel	2000-2001
4	12902369	Bilukha OO, et al^30^	2003	Afghanistan	2001-2002
5	15019123	Vassilia K, et al^31^	2004	Greece	1996-2000
6	15798470	Amir LD, et al^32^	2005	Israel	2000-2002
7	16818578	Witsaman RJ, et al^33^	2006	USA	1990-2003
8	16882957	Bilukha OO, et al^34^	2006	Chechnya	1994-2005
9	16953022	Coppola CP, et al^35^	2006	Iraq	2004-2005
10	18711835	Bilukha OO, et al^36^	2007	Chechnya	1994-2005
11	17208567	McGuigan R, et al^37^	2007	Iraq	2004
12	19557963	Bilukha OO, et al^38^	2008	Afghanistan	2002-2006
13	18977963	Matos RI, et al^39^	2008	Iraq	2003-2004
14	19782317	Can M, et al^40^	2009	Turkey	2002-2007
15	19820583	Creamer KM, et al^41^	2009	Iraq, Afghanistan	2002-2007
16	19838104	Jaffe DH, et al^42^	2010	Israel	2000-2005
17	21296800	Bilukha OO, et al^43^	2011	Nepal	2006-2010
18	23117384	Edwards MJ, et al^44^	2012	Iraq, Afghanistan	2002-2010
19	22524930	Arul GS, et al^45^	2012	Afghanistan	2011
20	24099375	Bagri N, et al^46^	2013	India	2010-2011
21	24553560	Edwards MJ, et al^47^	2013	Iraq, Afghanistan	2002-2010
22	24650471	Villamaria CY, et al^48^	2014	Iraq, Afghanistan	2002-2011
23	26116000	Hillman CM, et al^49^	2014	Afghanistan	2003-2014
24	24307254	Inwald DP, et al^50^	2014	Afghanistan	2011-2012
25	26374671	Mousavi B, et al^51^	2015	Iran	1988-2014
26	26165650	Çelikel A, et al^52^	2015	Syria	2012-2014
27	26692453	Khan I, et al^53^	2015	Pakistan	2010-2011
28	27483523	Bitterman Y, et al^54^	2016	Israel	2013
29	27550873	Billock RM, et al^55^	2016	USA	1990-2014
30	27679958	Myers J, et al^56^	2016	USA	2006-2012
31	27530971	Ashkenazi I, et al^57^	2016	Israel	1994-2005
32	27771222	Er E, et al^58^	2017	Syria	2013-2014
33	29908849	El Chehab H, et al^59^	2018	Afghanistan	2009-2012
34	31739348	Çelikkaya ME, et al^60^	2020	Syria	2011-2019
35	30902455	Naaman O, et al^61^	2020	Syria	2013-2016
36	29055895	Thompson DC, et al^62^	2020	Afghanistan	2006-2013
37	31676974	Bäckström F, et al^63^	2020	Iraq	2017
38	33308824	Marenco CW, et al^64^	2021	Iraq, Afghanistan	2008-2015

### Data Quality

Quality assessment was performed utilizing an 11-point MMAT grading tool due to its ability to successfully evaluate quantitative non-comparative studies. Overall, the mean score attained was 8.81 (SD = 2.4) out of a possible 11.0 points (Supplementary Material, Table S2; available online only). The lowest score attained by this grading scheme was a two (n = 1), while the highest score attained was an eleven (n = 16). No studies attained a score of three and two studies attained a score of four. Overall, this suggested that the majority of the studies included in this work were of high quality and were thus feasible to extract and collate data from.

### Study Characteristics

**Figure 2.**
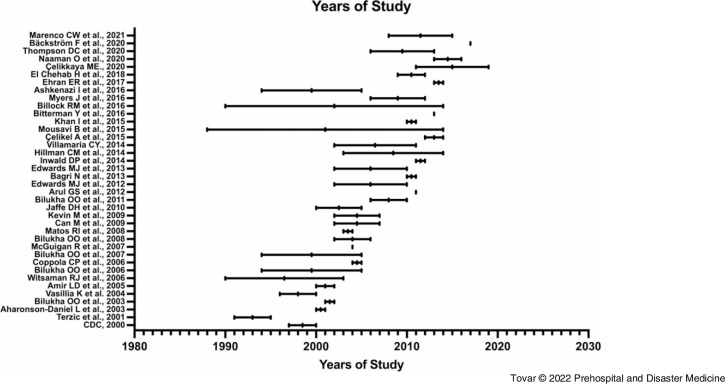
Years of Conflict Covered by Each Representative Study Included in the Meta-Analysis.

The majority of the articles (n = 33/38; 84.8%) from which data were extracted were retrospective cohort in design, followed by database search (n = 3/38; 7.9%), cross-sectional (n = 2/38; 5.3%), and prospective observational studies (n = 1/38; 2.6%). Most studies (n = 34/38; 89.4%) described explosions occurring outside the continental United States (OCONUS) and the minority (n = 5/38; 13.1%) described explosions that occurred within the continental United States (CONUS). Of studies that described OCONUS events (n = 34), 18/34 (52.9%) were related to the Global War on Terror (GWOT) campaign and 16/34 (47.0%) were unrelated to the GWOT campaign. Of 18 studies describing non-terrorism events, 6/18 (33.3%) were related to firework explosions and 7/18 (38.9%) were related to land mine explosions left over from prior conflicts. The remaining 5/18 studies (27.8%) described explosions produced by state-sponsored entities (eg, the Syrian Arab Republic utilizing ordnance that results in civilian casualties in the on-going Syrian civil war).^65^ The study pool described conflicts and accidents ranging from the years 1988-2017 (Figure 2).

### Patient Demographics

The 38 articles from which data were extracted collectively described 128,424 pediatric patients affected by blast injuries, sustaining a total of 222,638 unique recorded injuries (19,879 injuries related to terrorism and 202,759 injuries unrelated to terrorism). Of this study population, 9,962 patients were affected by explosions related to terrorism and 118,462 patients were affected by explosions unrelated to terrorism. All data analyzed passed all metrics for normal data distribution. The demographic data (Table 2) illustrated that the average age of blast victims affected by bombings related to terrorism was lower than blast victims affected by non-terrorism explosions (10.28 years-old, SD = 2.7 versus 12.07 years-old, SD = 1.87, respectively). The biological sex distribution was also different between terrorism victims versus accidental explosion victims (68.96% male, SD = 17.58 versus 79.15% male, SD = 8.02, respectively**)**. The overall mortality rate of explosions related to terrorism was 15.4% (SD = 19.3%) compared to 5.2% (SD = 7.1%) of explosions unrelated to terrorism.

**Table 2.** Demographics of Victims of Explosions Related-To and Unrelated-To Terrorism

Demographic	Terrorism-Related	Terrorism-Unrelated
Age	10.3 years (SD = 2.7)	12.1 years (SD = 1.8)
Biological Sex		
Male	68.96% (SD = 17.84)	79.15% (SD = 8.02)
Female	31.04% (SD = 8.98)	20.85% (SD = 4.02)
ISS	16.33 (SD = 3.29)	–

Note: Compared using Student’s t-test with Welch’s correction for unequal variance with P < .05 considered statistically significant.

Abbreviation: ISS, Injury Severity Score.

### Risk Factors for Pediatric Mortality

**Figure 3.**
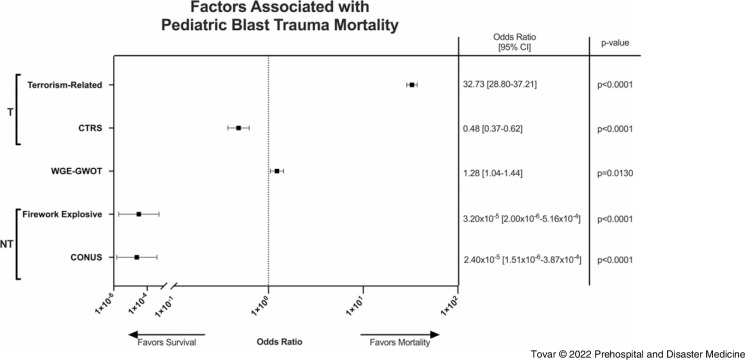
Bivariate Odds Ratio Analysis Revealed Several Protective and Harmful Factors Related to Pediatric Mortality. Note: Overall, explosions related to terrorism (T) were highly associated with pediatric mortality compared to explosions unrelated to terrorism (NT). However, being resuscitated at a forward-deployed CTRS was a protective factor against pediatric mortality compared to those who were not resuscitated at a CTRS. Use of WGEs in conflicts related to the GWOT associated with increased odds of mortality when compared to use of WGEs in conflicts not associated with the GWOT. Finally, for NT explosions, protective factors included if the event took place in the CONUS and if a commercial firework was the explosive.Abbreviations: CTRS, combat trauma resuscitation service; WGE, weapons-grade explosive; GWOT, Global War on Terror; CONUS, Continental United States.

Bivariate odds ratio analyses of situational factors surrounding the setting of the explosion showed several harmful and protective factors associated with pediatric mortality (Figure 3). The odds of mortality were significantly higher in the setting explosions related to terrorism compared to those unrelated to terrorism (OR = 32.73; 95% CI, 28.8-37.2; P < .0001). Weapons-grade explosives (WGEs), including improved explosive devices (IEDs), land mines, unexploded ordinance, and conventional munitions, detonated in GWOT conflicts were also associated with increased odds of pediatric mortality compared to WGEs detonated in non-GWOT conflicts (OR = 1.28; 95% CI, 1.04-1.44; P = .0130). Being treated at a North Atlantic Treaty Organization (NATO; Brussels, Belgium)-affiliated combat support hospital in the GWOT was associated with decreased pediatric mortality in the setting of terror-related explosions (OR = 0.48; 95% CI, 0.37-0.62; P < .0001). With respect to explosions unrelated to terrorism, the odds of mortality were substantially lower if the explosive agent was a commercial firework compared to accidental detonation of landmines and unexploded ordnance (OR = 3.20×10^-5^; 95% CI, 2.00×10^-6^-5.16×10^-4^; P < .0001). Victims of explosions unrelated to terrorism also had decreased odds of mortality if the incident occurred within CONUS compared to OCONUS (OR = 2.40×10^-5^; 95% CI, 1.51×10^-6^-3.87×10^-4^; P < .0001).

### Pediatric Injury Profiles and Injury Severity

**Figure 4.**
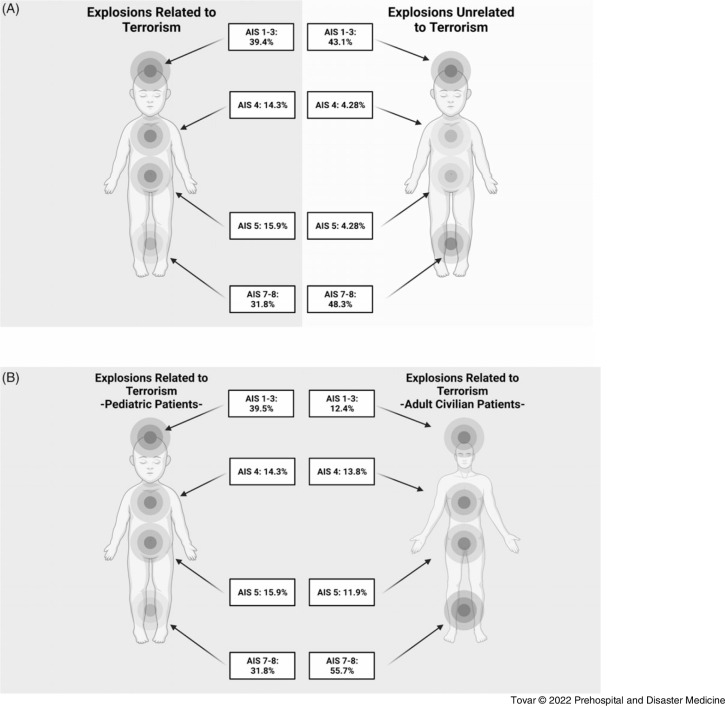
**(A)** Pooled Summary of Pediatric Injury Profiles in Explosions Related to Terrorism and Unrelated to Terrorism; and **(B)** Pooled Summary of Pediatric Injury Profile Compared to Adult Civilian Injury Profile in Blasts Related to Terrorism. Abbreviation: AIS, Abbreviated Injury Scale.

There were differences in the injury profile between those affected by terror-related blast trauma versus those affected by non-terror-related explosions (Figure 4A). When stratified by injury region, there was a similar incidence of head, face, and neck trauma (AIS 1-3) between terrorism and non-terrorism explosions (39.5% versus 43.1%, respectively). However, there was an increase in the incidence of thoracic injury (AIS 4; 14.3% versus 4.28%) and abdominal injury (AIS 5; 15.9% versus 4.3%) in explosions related to terrorism compared to explosions unrelated to terrorism. Finally, the incidence of extremity injury (AIS 7-8) was increased in explosions unrelated to terrorism compared to explosions related to terrorism (48.3% versus 31.8%, respectively). It was also noted that the pediatric blast injury profile was unique compared to the adult civilian blast injury profile (Figure 4B) with a higher incidence of injury to AIS 1-3 but a lower incidence of injury to AIS 7-8 in the pediatric population compared to adults.^66^ Only one non-terror-related trauma article reported victim ISS, and thus the ability to compare this reported score to terror-related blast victims was unachievable. The average ISS among terror-related blast victims was 16.0 (SD = 6.30). Within explosions related to terrorism, there was a statistically significant increase in the ISS of those mortally wounded versus those non-mortally wounded (44.5, SD = 22.1 versus 16.5, SD = 5.47; P = .006).

## Discussion

### Understanding Differences in Pediatric Injury Profile between Explosions Related to Terrorism versus Unrelated to Terrorism

This study presents the first meta-analytical study of environmental risk factors of pediatric mortality in the setting of explosions related and unrelated to terrorism. Fundamental differences were noted in the injury patterns of victims affected by explosions related to terrorism compared to those affected by explosions unrelated to terrorism. Victims affected by explosions related to terrorism had a higher incidence of thoracoabdominal trauma, a lower incidence of extremity trauma, and a similar incidence of craniocerebral trauma compared to victims affected by explosions unrelated to terrorism. These findings are likely explained by the observation that most articles that described non-terror explosions involved a patient either accidentally stepping on old, unexploded ordnance or holding an explosive in their hand, thus inducing a higher level of extremity injury than explosions related to terrorism. The higher incidence of thoracoabdominal trauma in explosions related to terrorism likely stems from the use of higher quantities of high-grade explosives in bombings compared to what is present in unexploded ordnance or low-grade firework explosives.^67–69^

Furthermore, it was found that the use of WGEs in GWOT conflicts were associated with increased mortality compared to both WGEs not used in GWOT conflicts and non-WGE devices (eg, fireworks). A practical example of this delineation is that IEDs constructed and detonated by a terrorist cell are more likely to increase mortality compared to anti-personnel mines left over from a conflict accidentally triggered to detonate. This is likely related to the fact that the amounts of explosive used in unexploded ordnance is much smaller than that used in modern-day terrorism bombings. Unexploded ordnance typically contain between 1-30kg of explosive materials while modern-day IEDs have been reported to have between 9kg to >4,000kg of explosive maternal.^9,70^ Explosions related to terrorism also typically use high-grade explosives such as nitroglycerin,^71^ plasticized cyclonite,^72,73^ peroxide-based explosives,^74^ and ammonium nitrate^75^ where the velocity of the blast pressure front is many times greater than the velocity produced by explosives in commercial-grade fireworks (approximating 4500m/s versus less than 1000m/s, respectively).^76,77^ Furthermore, terrorist groups have been reported to utilize a combination of directional amplifiers to further increase the velocity of the blast pressure wave and metal foreign bodies designed to maximize penetrating trauma, further improving control of the pressure wave and the lethality of the explosive, respectively.^77,78^ This directional concentration of the blast pressure wave from explosives used in GWOT conflicts further increases the potential to cause devastating injury and mortality compared to other WGEs and commercial explosives.

A lower odds of pediatric victim mortality in casualties treated at NATO-affiliated hospitals was also discovered at forward-deployed combat resuscitation services compared to non-NATO affiliated civilian hospitals. This is potentially due to superior trauma resuscitation practices of forward-deployed NATO combat resuscitation services, as trauma casualties sustained during the GWOT revolutionized the science of traumatology. During the GWOT, a major revision in resuscitative practices resulted in a decrease in the mortality rate of injuries sustained in combat.^79,80^ Examples of such practices include the wide-spread adoption of tourniquet use to stop exsanguinating hemorrhage, increased use of walking blood banks, use of tranexamic acid to promote hemostasis, and aggressive forward damage control resuscitation.^81^ These resuscitative guidelines likely contributed to the lower mortality rate of pediatric patients in patients resuscitated at NATO combat resuscitation hospitals compared to those not treated at these facilities. However, to date, there is no unified combat trauma treatment protocol to guide medical personnel in the treatment of pediatric trauma sustained in the setting of armed conflict as there is in the adult population.^82^ Further research in this space is required to further minimize death and morbidity in the pediatric population.

Although the annual incidence of injury from fireworks contributes to the largest explosion-related injury mechanism in the United States, the incidence of pediatric fatality is extremely low.^10^ A federal government report not captured by the systematic review protocol recorded only one pediatric fatality associated with a fireworks explosion in 2020, which saw upwards of 15,600 fireworks-related injuries across the country.^10^ The low observed mortality rate likely stems from the fact that firework explosives in the United States are regulated by both state and federal statutes (Regulation 27 CFR Part 555 promulgated by the Bureau of Alcohol, Firearms, and Tobacco [ATF; Washington, DC USA]) that are designed to keep firework-related mortality low. Nevertheless, isolated case studies of firework factory explosions that caused severe morbidity and mortality in all ages (including children) have been reported in the literature but were either not captured or excluded by this systematic review protocol.^83–85^

### Differences in Pediatric versus Adult Injury Profile in the Setting of Explosions Related to Terrorism

The injury patterns of pediatric blast victims is significantly different from a cohort of adult blast trauma patients previously studied in a similar manner.^66^ The incidence of craniocerebral trauma in the pediatric population (n = 7,860 injuries to AIS 1-3 out of 19,879 total injuries, incidence = 39.5%) was approximately 30% higher than their civilian adult counterparts (1,845 injuries to AIS 1-3 out of 14,676 total injuries, incidence = 12.5%) in the setting of trauma sustained from a terrorism bombing. Also, the incidence of extremity trauma was reduced by upwards of 36% in pediatric patients (6,336 injuries to AIS 7-8 out of 19,926 total injuries, incidence = 31.8%) when compared to civilian adults (9,924 injuries to AIS 7-8 out of 14,676 total injuries, incidence = 67.6%) in the setting of trauma sustained from a terrorism bombing.^66^ These differences in injury patterns are suspected to be largely associated with body surface area of a given pediatric patient compared to their adult counterpart as the volume and surface area ratio of the craniocerebral structures to the rest of the body is significantly larger in the pediatric patients compared to adults.^86^ In addition, the presence of open cranial fontanelles in infants and toddlers also have the potential to contribute to an increased incidence of traumatic intracranial pathology, as has been shown in biomechanical modeling studies.^87,88^

### Unique Utility of Injury Profile Data: Advantages of Designing Data-Driven Interventions to Mitigate Disaster Morbidity and Mortality

It was posited that injury profile meta-analyses such as the one prepared here have the potential to influence emergency medical provider education—both prehospital and in-hospital. For example, emergency medical response agencies can use the finding of increased head trauma in pediatric patients compared to adults to focus on screening for head trauma in the field and transporting patients to pediatric trauma centers with neurosurgical capabilities. Alternatively, if providers live in an area prone to terrorist attacks (ie, in war zones, areas of violent civil conflict, or highly targeted cities like Washington, DC [USA], New York City [New York USA], or London, United Kingdom), there is a need to be prepared for complex craniocerebral and thoracoabdominal trauma in the pediatric population.

These injury profile data can also be successfully leveraged when designing disaster response packages utilizing the “all-hazards approach” philosophy. When designing response packages, the all-hazards approach philosophy dictates that emergency preparedness be focused on ensuring capacities and capabilities critical to respond to a wide variety of disasters. Leveraging these data could potentially involve assigning more fiscal and personnel resources dedicated to addressing pediatric head and chest trauma. Examples of such resource allocation strategies include increasing the availability of staff capable of performing emergent cranial decompressions and ensuring robust access to pediatric thoracotomy surgical trays during times of heightened terrorism threats. Organizations utilizing a data-driven approach for the organization of emergency medical response protocol can optimize their life-saving efficacy while minimizing over-spending and resource wasting with the accumulation of unnecessary supplies.

## Limitations

A number of studies (15/39; 38.4%) extracted data from hospital records, not standardized trauma databases, potentially introducing a degree of data heterogeneity since retrospective chart reviews are not as uniform as trauma database inputs. In addition, many papers were excluded from the study solely due to the fact that pediatric patient injury profiles were not separated from adult patients. Moreover, markers of pediatric morbidity, such as ISS, were inconsistently reported and thus were not able to be compared across cohorts of blasts related versus unrelated to terrorism. Due to the nature of disaster epidemiology papers reporting a large number of patients, it was difficult to ascertain the severity of a given patient’s injuries. As a result, any given “head injury” code might have been a simple scalp laceration requiring routine wound care, or it could have been a devastating injury requiring surgical intervention. As it was not reported in the study details, the authors were not able to provide further detail or compare injury severity, particularly with respect to craniocerebral injury.

## Conclusion

The first epidemiological study that describes mortality risk factors and the blast injury profile of pediatric victims is reported in this work. Moreover, novel differences in the blast injury pattern of patients exposed to explosions related to terrorism versus explosions unrelated to terrorism were reported as well. Pediatric victims of explosions related to terrorism had a higher incidence of thoracoabdominal trauma and a lower incidence of extremity trauma when compared to victims of explosions unrelated to terrorism. Emergency response agencies should use these data to optimize disaster education and disaster response packages with the goal of further reducing pediatric morbidity and mortality in the setting of acts of terrorism.

## Supplementary material

For supplementary material accompanying this paper visit https://doi.org/10.1017/S1049023X22000747. click here to view supplementary material
